# Enhancements in morphology, biochemicals, nutrients, and L-Dopa in Faba bean through plant growth promoting rhizobacteria and arbuscular mycorrhizal Fungi

**DOI:** 10.1038/s41598-025-92486-8

**Published:** 2025-03-03

**Authors:** Hilal Yilmaz

**Affiliations:** Plant and Animal Production Program, Izmit Vocational School, Kocaeli University, Kocaeli, 41285 Turkey

**Keywords:** Biostimulants, Chlorophyll, DPPH, L-Dopa, Phenolic content, Plant development, Plant physiology, Plant symbiosis, Secondary metabolism

## Abstract

Faba beans are among the main food sources grown since ancient times; seeds contain higher protein (23–41%) than other legumes and are rich in healthy secondary bioactive components. This study was carried out to determine the effects of plant growth-promoting rhizobacteria (PGPR) and arbuscular mycorrhizal fungi (AMF) on the biofortification potential of faba beans. The study utilized *Bacillus subtilis*, *Bacillus megaterium*, and an AMF mix to examine changes in growth parameters, chlorophyll content, bioactive compounds, and mineral nutrients. AMF and PGPR treatments significantly enhanced various traits in faba bean pods compared to untreated control plants. *Bacillus subtilis* treatment increased the antioxidant capacity by 128% and the total phenol content by 230%. AMF inoculation elevated the L-Dopa content, an essential compound for Parkinson’s disease prevention, by 92%. Protein content increased by 51% with *Bacillus megaterium* inoculations. The macro and micronutrients exhibited the highest enhancement in AMF treatment (Ca; 33.6%, K; 49.4%, Mg; 22.7%, P; 10.5%, Fe; 37.7%, Zn; 24.5%). The study results suggest that AMF and PGPR inoculation to faba bean seeds improves the morphological characteristics, bioactive compounds, and mineral nutrient content and is an excellent tool for obtaining biofortified faba beans through sustainable practice.

**Supplementary Information:**

The online version contains supplementary material available at 10.1038/s41598-025-92486-8.

## Introduction

The Faba bean (*Vicia faba* L.) is one of the oldest legumes in the Leguminosae family and has been a staple food for humans since ancient times^1^. It is widely adapted to various ecological conditions, from the polar circle to the equator and from high altitudes to sea level^2^. In 2022, faba bean cultivation worldwide was carried out on a total area of 2.68 million hectares, and 6.14 million tons of products were obtained^3^. The faba bean, grown in China, Ethiopia, Australia, and the UK, is a cheap daily protein source for North Africa, the Middle East, West and South Asia, and East African countries^4^.

Faba beans have a low-fat content, low glycemic index, and are rich in carbohydrates, vegetable protein, iron, zinc, and dietary fiber^5^. Its seeds have more protein (23–41%) than other legume plants^6^. It is an important source of essential amino acids such as arginine, lysine, and leucine^4^. It is also rich in secondary bioactive compounds such as antioxidants and phenols that benefit human health^7^, particularly the compound L-3,4-dihydroxyphenylalanine (L-Dopa), which is found in drugs that have been used successfully in the treatment of Parkinson’s disease for more than 50 years^8,9^ Faba beans can be a medicinal plant due to their beneficial contributions to Parkinson’s patients^10^. Synthetic dopamine drugs are not affordable and accessible to low-income people, especially in underdeveloped countries^11^. Approximately 2 billion people worldwide suffer from hidden hunger, primarily due to iron, zinc, and vitamin deficiencies^2,12^. One of the formulas developed to combat hidden hunger is the development of food varieties with increased levels of bioavailable mineral micronutrients, called biofortification^13^. The faba bean is an affordable and accessible natural source of L-Dopa^14^. Thanks to its rich bioactive compounds, it can be utilized as a functional food source in treating or preventing some diseases^15^. In addition, the faba bean is a strategic plant that could be a substitute for meat for people living in poor countries as a cheap source of protein and minerals^2,16^.

Modern agriculture is undergoing significant transformations due to escalating environmental challenges driven by the rapid growth of the global population^17^. This demographic expansion contributes to nutritional deficiencies in food supplies^18^, while shifting climatic conditions reduce crop yields and exacerbate food insecurity^19^. Consequently, redefining agricultural management and production strategies has become imperative to ensure economic sustainability^20^. In countries with agriculture-based economies, increasing yield per unit area is crucial for meeting food and nutritional demands while maintaining financial stability^19^. However, higher production levels alone do not guarantee improved nutritional outcomes; food quality is equally vital. In this context, one of the most pressing challenges in modern agriculture is maximizing food production and enhancing its nutritional value^21^. Biofortification is a key strategy to improve both yield and nutritional quality in crops^22^. Biostimulants, such as root bacteria and fungi, enhance plant growth, nutrition, and stress resistance^23,24^. Phosphorus and micronutrient transition metals (Fe, Cu, Mn, and Zn) are poor in alkaline soils^25^. Plant growth-promoting rhizobacterium (PGPR) increase soil nitrogen productivity by colonizing the rhizosphere, producing siderophore, and playing a role in the chelation of Fe ions and the solubility of minerals that are difficult to absorb, such as P^26^. In addition, microbial biostimulants act on plants’ primary and secondary metabolism, leading to the synthesis and accumulation of secondary metabolites such as antioxidant molecules that are important in human nutrition^24^. Some studies have evaluated the sole effects of AMF and PGPR on the yield parameters of faba beans^25,27^. However, these bioinoculants were not compared in a single to study compare their effects on chlorophyll, phenolic, and L-dopa contents in faba beans. Considering the above literature, this study aimed to evaluate the effects of AMFs and PGPRs on bioactive compounds, the accumulation of macro- and micro-nutrients, and some morphological characteristics in faba beans. In particular, the effects of PGPR and AMF on L-Dopa in faba beans were revealed for the first time in this study.

## Materials and methods

### Plant material and field experiment

The “Sorgun” Turkish faba bean variety obtained from Küçük Çiftlik Tohumculuk company (https://kucukciftlik.com) was used as plant material. The experiment was carried out under Bolu ecological conditions (40°41’12.0"N, 31°35’28.9"E) in 2022. The study was constituted of four treatments (*Bacillus subtilis*, *Bacillus megaterium*, AMF mixture, and control). Faba bean seeds were sown into a 3.6 m^2^ (2 m x 1.8 m) plot for each treatment with 30 seeds per m^2^, with 50 cm and 20 cm between and within rows on April 20. The block and plot spacings were 2 and 1 m, respectively. The harvest was conducted manually during the first week of September when the pods were young, tender, and completely fulfilled.

The climate data for 2022 and long-term average (last 20 years) for the Bolu province, where the trial land is located, are given in Table 1. The monthly average temperature during the growth season (April–September) was between 9.7℃ and 16.1℃. More rainfall was in April, May, and June than in other months. Comparable in monthly distribution to monthly precipitation, the average relative humidity ranged from 69.2% in August to 72.1% in June. In 2022, the monthly temperatures closely matched the long-term distributions with a little variance (± 1). The precipitation regime and relative humidity remained well below the long-term trend except for June and August. June saw the most rainfall (163.3 mm), with September having the lowest rainfall (10.0 mm). Properties analyses of trial soil were conducted in the AB-0299 test laboratory accredited by TÜRKAK (Turkish Accreditation Agency), and the data are presented in Table 2.

**Table 1:** The climate data belonging to the Bolu Province. *TR* total rainfall, *LT* long term, *RH* relative humidity, *AT* average temperature.

Parameter	April	May	June	July	August	September
TR in 2022 (kg/m^2^)	29.0	39.0	163.0	20.0	54.0	10.0
LT TR (kg/m^2^)	51.1	62.5	63.2	28.9	27.5	27.5
RH in 2022 (%)	65.0	66.0	79.0	70.0	75.0	69.0
LT RH (%)	70.0	71.7	72.1	69.5	69.2	71.1
AT in 2022 (℃)	10.0	13.0	17.0	19.0	21.0	16.0
LT AT (℃)	9.7	14.0	17.3	19.8	19.8	16.1

**Table 2:** Soil properties of the experimental field. *P*_*2*_*O*_*5*_ phosphorus pentoxide, *EC* electrical conductivity, *N* nitrogen, *Ca* calcium, *K* potassium, *Mg* magnesium, *Cu* copper, *Mn* manganese, *Zn* zinc, *Fe* iron.

Physical Properties	Value	Exchangeable cations (mg kg^− 1^)	Value
Texture	Clay Loam	Ca	6309
Organic carbon (%)	2.70	K	1040
P_2_O_5_ (kg da^− 1^)	45.01	Mg	763.1
EC (dS m^− 1^)	0.76	Cu	3.62
pH	7.22	Mn	6.23
N (%)	0.15	Zn	2.41
Lime (%)	1.63	Fe	4.99

### Microbial applications

The *Bacillus subtilis* (DSM 23778) and *Bacillus megaterium* (ATCC 14581) bacterial strains were obtained from Prof. Dr. Metin Turan (Yeditepe University, Turkey) as ready-to-use solution kept in a cold-chain. AMF mixture was obtained from Bioglobal^®^ company (Antalya, Turkey) in powder form. Bacterial cultures grown on nutrient agar were kept at + 4 ℃ until inoculation. The bacterial application was performed by modifying the method proposed by Yılmaz and Kulaz^28^. Sixty seeds were inoculated with 10 ml bacterial solution containing 10^8 CFU ml^− 1^ concentration before sowing.

The Endo Roots Soluble (ERS) mixture package includes *Rhizophagus irregularis* (21%), *Funneliformis mosseae* (20%), *Glomus aggregatum* (20%), *Glomus monosporum* (1%), *Glomus deserticola* (1%), *Paraglomus brasilianum* (1%), *Glomus etunicatum* (1%), *Glomus clarum* (1%) and *Gigaspora margarita* (1%) fungi. The package contains fungus with a guaranteed total live organism presence of 23.5%. The AMF mixture inoculation process was carried out with a solution prepared by mixing 25 g of ERS in 1 L of distilled water^29^. The solution consists of approximately 250,000 spores. A hundred seeds were infected with a 10 ml solution, which accounts for 25 spores per seed.

### Determination of growth parameters and mineral composition

Plant height (cm), pod weight (g), pod length (cm), and seed weight (g) were determined as plant growth parameters. The examined parameters were obtained from 20 plants randomly selected from each plot. One gram of ground faba beans was soaked overnight in a nitric acid (3 ml) solution for mineral element analysis. The next day, the samples were pre-burned at 200 ℃ for 1 h. 1 ml perchloric acid was added to the cooled mixture. The wet digestion process continued until the solution became clear. After the wet digestion process, the solutions were filtered using filter paper, and the final volumes were completed to 25 ml with distilled water. K, Ca, Mg, Fe, and Zn analyses were determined using an Atomic Absorption Spectrophotometer (Thermo Scientific/ICE 3000) except for phosphorus analysis (ICP-OES/Thermo Scientific/USA).

### Preparation of plant materials for analysis

The collected fresh faba bean pods were stored in a deep freezer at -20 ℃ until biochemical analyses were performed. Before the analysis, 5 g of faba bean pods from each sample were homogenized in 10 ml methanol. Then, the homogenates were centrifuged at 15,000 rpm for 10 min and the supernatant was separated and used in subsequent analyses.

### Determination of DPPH activity, total phenolic, total protein and L-dopa

The DPPH scavenging activity was determined using 2,2-Diphenyl-1-picrylhydrazyl (Sigma-Aldrich, Darmstadt, Germany), following the methodology outlined by Güler and colleagues^30^. A final absorbance range of 0.7–0.8 was used for analysis. The optimum amount of methanolic extract was determined by preliminary trials and the protocol was adjusted to a final volume of 2 ml. In the analysis, a 50 µl sample, 1.45 ml ethanol, and 0.5 ml DPPH solution were added to the tube in that order and vortexed. After 15 min, the solution’s absorbance was recorded at 520 nm with a UV-Vis spectrophotometer. The DPPH scavenging capacity was determined by calculating the change in absorbance (A) compared to the blank, which consisted of 1500 µl of ethanol and 500 µl of DPPH solution, using the following formula:

DPPH (%) = (A_blank_ − A_sample_)/A_blank_ x 100.

Total phenolic content (TPC) was determined using a modified method of Waterhouse’s^31^. Briefly, 1.6 ml of distilled water, 50 µl of methanolic extract, and 50 µl of Folin-Ciocâlteu agent were combined and gently mixed. 300 µl of calcium carbonate solution (7% w/v) was added to the mixture. After vortexing, the mixture was kept at room temperature in the dark for 2 hours and the absorbance of the mixture was measured at 760 nm using a UV-Vis spectrophotometer (SP-UV1100, DLAB, Beijing, China). For the standard curve (R^2^ = 0.99), the absorbance of solutions prepared with 0.5, 1, 2, 3, 4, 5, and 6 mM gallic acid was analyzed with the same procedure. The crude protein content of ground faba beans was determined using the Kjeldahl method^32^. Total protein values (%) were calculated by multiplying the obtained values by 6.25 (*N* × 6.25). L-Dopa was quantified by the method of Patil et al.^33^ with slight modifications. Briefly, 2 ml of %0.2 ninhydrin was added to 5 ml of diluted supernatant in heating tubes. The mixture was incubated at 80 ℃ for 5 min in a water bath. Then, it was transferred to test tubes, and the final volume was completed to 10 ml with dimethylformamide (DMF). A standard curve was created with 10, 20, 30, 40, and 50 µg/ml L-Dopa standard solutions following the same procedure. The absorbance was read at 632 nm by a UV-Vis spectrophotometer and real values were calculated against the standard curve.

### Determination of chlorophyll-a, chlorophyll-b, and total chlorophyll

Total chlorophyll, chlorophyll a, and b were determined using the Arnon method^34^. A sample of 0.8 g of faba bean was extracted using 80% acetone. The mixture was filtered through Watman #1 filter paper. The absorbance of the supernatant was determined with a spectrophotometer at wavelengths of 663 and 645 nm. Chlorophyll contents were calculated using the formulas below;

Chlorophyll a (mg g^− 1^ FW) = (12.7 × A_663_ – 2.69 × A_645_) x V / 1000 x g.

Chlorophyll b (mg g^− 1^ FW) = (22.9 A_645_ – 4.68 × A_663_) x V / 1000 x g.

A represents absorbance, FW represents fresh weight, V represents the volume of the extract, and g represents the sample volume (mg).

### Data analysis and statistical evaluations

The research was conducted using a randomized block trial design with three biological replications. Statistical analyses of the obtained data were performed using R Studio. A one-way ANOVA test was employed to determine the differences among the groups. When significant differences were detected, LSD (Least Significant Difference) test was used with a significance level of *p* ≤ 0.05 to determine which groups differed. The relationships between characteristics and treatments were assessed using principal component analysis (PCA). Pearson’s pairwise correlations were analyzed using the “corrplot” package in R Studio^35^.

## Results

### Growth parameters

**Fig. 1:**
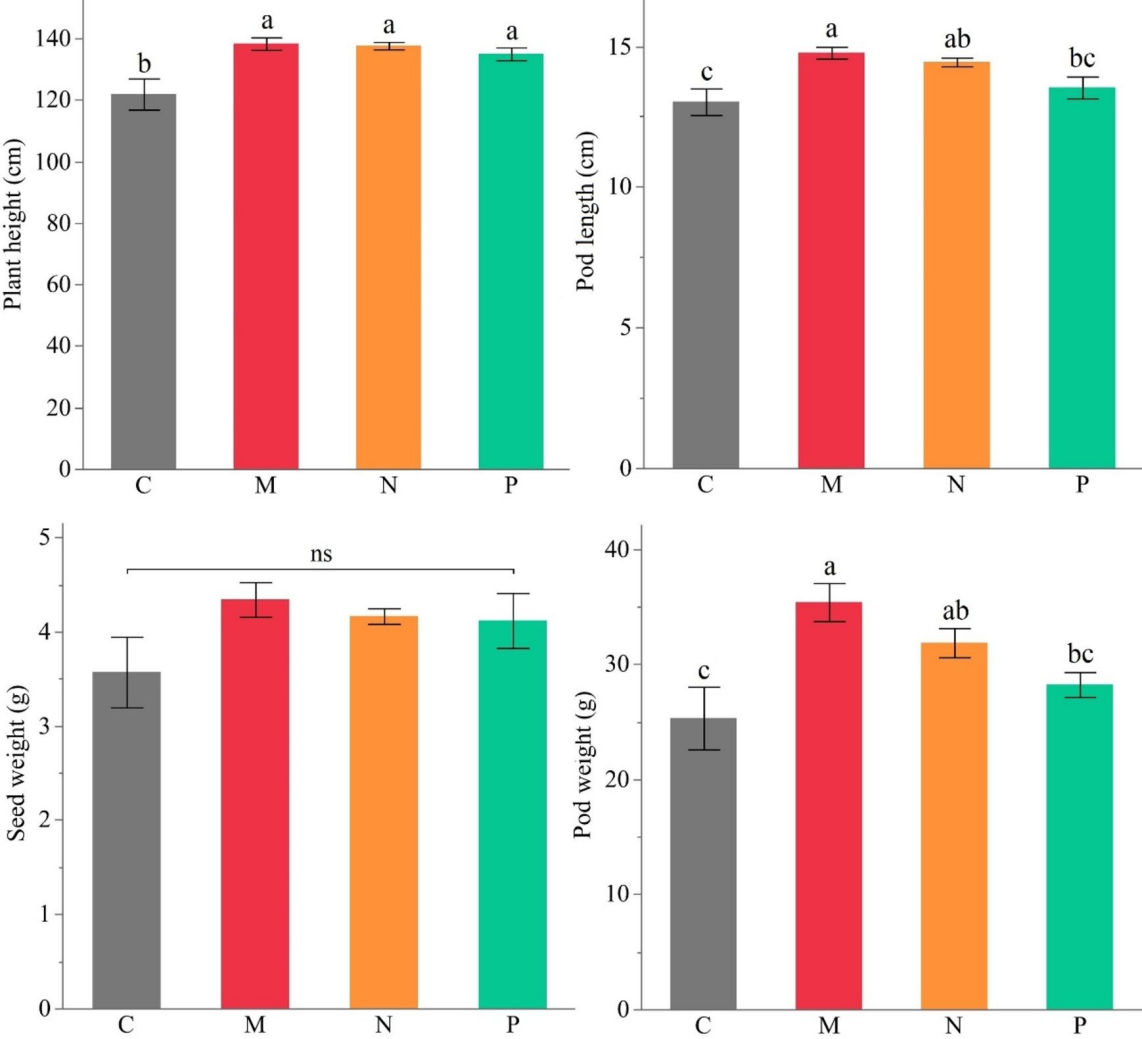
Effect of different AMF (M) and PGPRs (*Bacillus subtilis* (N) and *Bacillus megaterium* (P)) on plant height (cm), pod length (cm), pod weight (g), and seed weight (g) of faba bean (C: Control, Different letters indicate significant differences according to LSD test, ns: non-significant, * (*p* ≤ 0.05)).

The effects of mycorrhiza mixture, *Bacillus subtilis*, and *Bacillus megaterium* inoculations on plant height (cm), pod length (cm), pod weight (g) and seed weight (g), were evaluated (Fig. 1; Table S1). While significant differences were observed among applications for plant height, pod length and pod weight, no statistically significant variation was detected for seed weight.

Among the evaluated parameters, the application of AMF resulted in the most pronounced improvements, particularly in pod length and pod weight. Compared to the control, pod length increased by 13.4%, from 13.03 cm to 14.77 cm, while pod weight increased by 39.8%, from 25.33 g to 35.41 g. The treatments with *Bacillus subtilis* and *Bacillus megaterium* also contributed to pod length and pod weight enhancement; however, their effects were comparatively lower than AMF.

For plant height, although all treatments exhibited numerically higher values compared to the control, the differences were not statistically significant (*p* > 0.05). The mean plant height ranged from 121.93 cm in control to 138.33 cm in the AMF treatment. Similarly, no significant differences were detected in seed weight among the treatments. The recorded seed weights varied from 3.57 g in the control group to 4.34 g in the AMF-treated group.

### Chlorophyll-a, chlorophyll-b and total chlorophyll

The chlorophyll a, b, and total chlorophyll content of faba beans were significantly influenced by microbial inoculations, with *Bacillus subtilis* treatment showing the most substantial increase compared to the control. Specifically, chlorophyll a content increased from 1.91 mg g⁻¹ FW in the control to 3.10 mg g⁻¹ FW in *B. subtilis*-treated plants. Similarly, chlorophyll b content increased from 0.84 mg g⁻¹ FW to 1.19 mg g⁻¹ FW, and total chlorophyll content increased from 2.75 mg g⁻¹ FW to 4.29 mg g⁻¹ FW. These results indicate a statistically significant enhancement in chlorophyll accumulation under *B. subtilis* application.

The AMF and *Bacillus megaterium* treatments also led to increased chlorophyll levels compared to the control, but their effects were relatively moderate. Chlorophyll a content in AMF-treated plants reached 2.40 mg g⁻¹ FW, while *B. megaterium* treatment resulted in 2.37 mg g⁻¹ FW, both of which were significantly higher than the control but lower than *B. subtilis*. Chlorophyll b content in AMF (0.95 mg g⁻¹ FW) and *B. megaterium* (0.96 mg g⁻¹ FW) treatments did not show statistically significant differences from the control. Total chlorophyll content followed a similar pattern, with AMF and *B. megaterium* reaching 3.35 mg g⁻¹ FW and 3.33 mg g⁻¹ FW, respectively, both higher than the control but not significantly different.

**Fig. 2:**
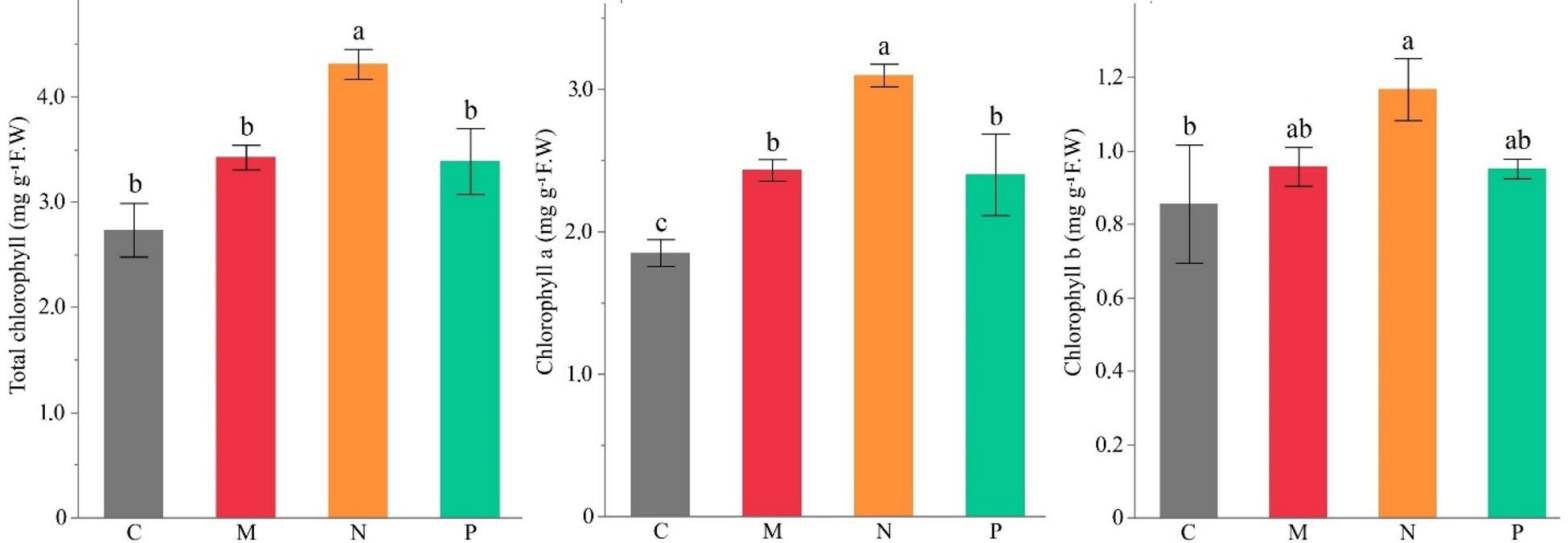
Effect of different AMF (M) and PGPRs (*Bacillus subtilis* (N) and *Bacillus megaterium* (P)) on chlorophyll a, b and total chlorophyll content of faba bean. (C: Control, Different letters indicate significant differences according to LSD test, * (*p* ≤ 0.05)).

Statistical analysis revealed that *B. subtilis* significantly enhanced all chlorophyll parameters compared to the control and other treatments, whereas *B. megaterium* and AMF only showed a significant increase in chlorophyll a but not in chlorophyll b or total chlorophyll content (Fig. 2; Table S2).

### L-Dopa, protein content, total antioxidant capacity, and total phenolic content

**Fig. 3:**
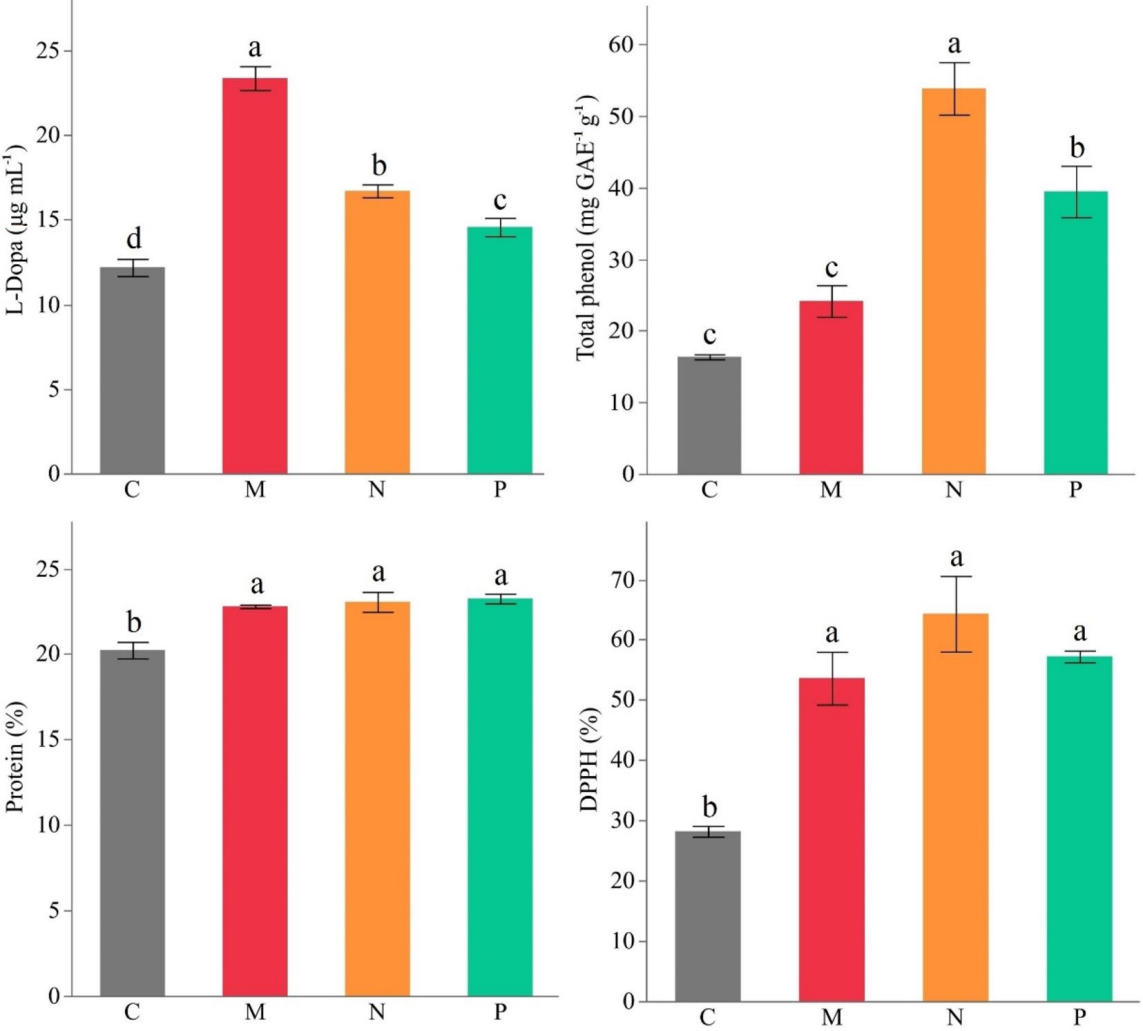
Effect of different AMF (M) and PGPRs (*Bacillus subtilis* (N) and *Bacillus megaterium* (P)) on L-Dopa, total antioxidant, total phenol, and protein content of faba bean (C: Control, Different letters indicate significant differences according to LSD test, * (*p* ≤ 0.05)).

In this study, L-Dopa was between 12.2 µg ml^− 1^ and 23.4 µg ml^− 1^ in the pods of faba beans. The difference between treatments was significant at *p* ≤ 0.05. The highest L-Dopa was determined as 23.4 µg ml^− 1^ in the AMF treatment, which was approximately 92% more than the control. The difference in protein content of faba beans is not statistically significant for PGPR and AMF applications but all treatments exhibited significantly enhanced protein content rather than control. The protein content increased by approximately 15% in the inoculated plants as compared with the control. The DPPH scavenging activity of the control treatment was 28%. The treatments possessed significantly higher DPPH scavenging activity than the control, but they did not significantly differ. The highest DPPH scavenging activity was detected in the *Bacillus subtilis* treatment with a 128% increase compared to the control (Fig. 3). The TPC of faba beans ranged from 16.3 mg^− 1^ GAE eq to 53.8 mg^− 1^ GAE eq. The lowest TPC was measured in control. The highest content was 53.8 mg^− 1^ GAE eq in *Bacillus subtilis* inoculation. PGPR and AMF applications increased the antioxidant capacity and TPC of faba beans (Fig. 3, Table S3).

### Mineral nutrient contents

**Fig. 4:**
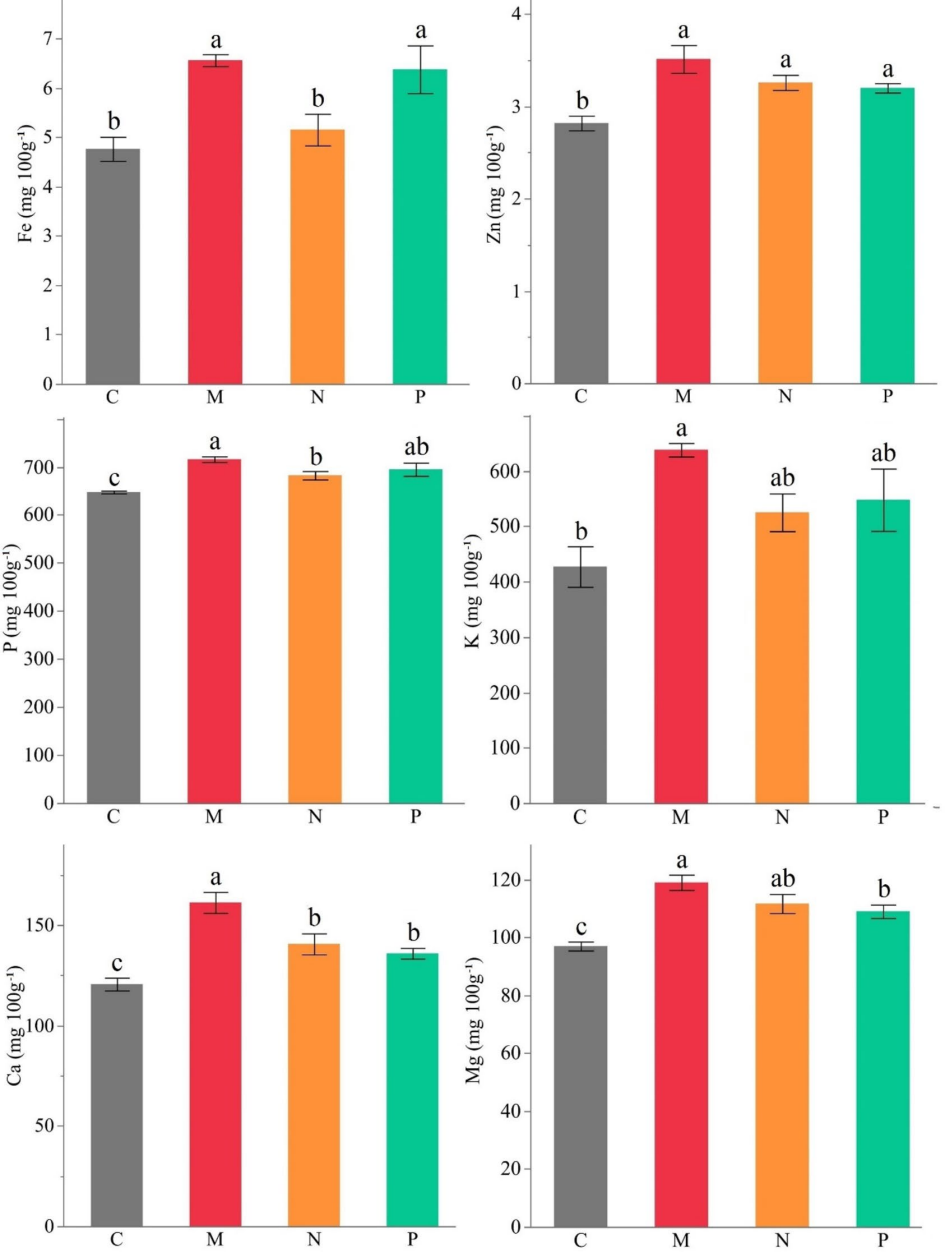
Effect of different AMF (M) and PGPRs (*Bacillus subtilis* (N) and *Bacillus megaterium* (P)) on Fe, Zn, P, K, Ca, and Mg (mg 100 g^− 1^) mineral nutrients of faba bean (C: Control, Different letters indicate significant differences according to LSD test, * (*p* ≤ 0.05)).

The highest Fe accumulation in faba beans was detected in the AMF (6.57 mg 100 g^− 1^) and *Bacillus megaterium* (6.38 mg 100 g^− 1^) treatments. The control comprised 4.77 mg/100 g iron and AMF increased iron accumulation in pods by 37.7%. All treatments exhibited significantly higher zinc accumulation than the control. The AMF, *Bacillus subtilis*, and *Bacillus megaterium* inoculations were not significantly different. The beans of control comprised 2.82 mg 100 g^− 1^ zinc and AMF increased content by 24.5% (3.51 mg 100 g^− 1^). The phosphorus was the richest element in the faba beans and accumulation in pods was higher than the control in all treatments. The control comprised 647.50 mg 100 g^− 1^ phosphorus followed by *Bacillus subtilis* (682.50 mg 100 g^− 1^) and *Bacillus megaterium* (695.00 mg 100 g^− 1^). The highest phosphorus content was determined in AMF inoculation as 715.80 mg 100 g^− 1^. Potassium accumulation exhibited the highest rational increase across the mineral nutrients in faba beans. The AMF treatment yielded significantly higher K than the control, while the other treatments were not significantly different. AMF-treated faba bean pods comprised 49.4% more K (638.33 mg 100 g^− 1^) compared to the control (427.33 mg 100 g^− 1^). Although the other treatments did not exhibit significant differences, K contents were comparably higher in *Bacillus subtilis* (525.33 mg 100 g^− 1^) and *Bacillus megaterium* (548.00 mg 100 g^− 1^) than the control. Calcium accumulation differed significantly across treatments, and all treatments exhibited significantly higher contents than the control. In control, an average of 120.7 mg 100 g^− 1^ Ca was detected in pods. The AMF caused 161.3 mg 100 g^− 1^ Ca accumulation in pods, which was 33.6% more than the control (120.67 mg 100 g^− 1^). Mg content was significantly higher than the control in all treatments. The Mg was 97 mg 100 g^− 1^ in the control, while it was 119 mg 100 g^− 1^ in the AMF, with an increment of 22.7% compared to the control (Fig. 4, Table S4).

### Principal component analysis

**Fig. 5:**
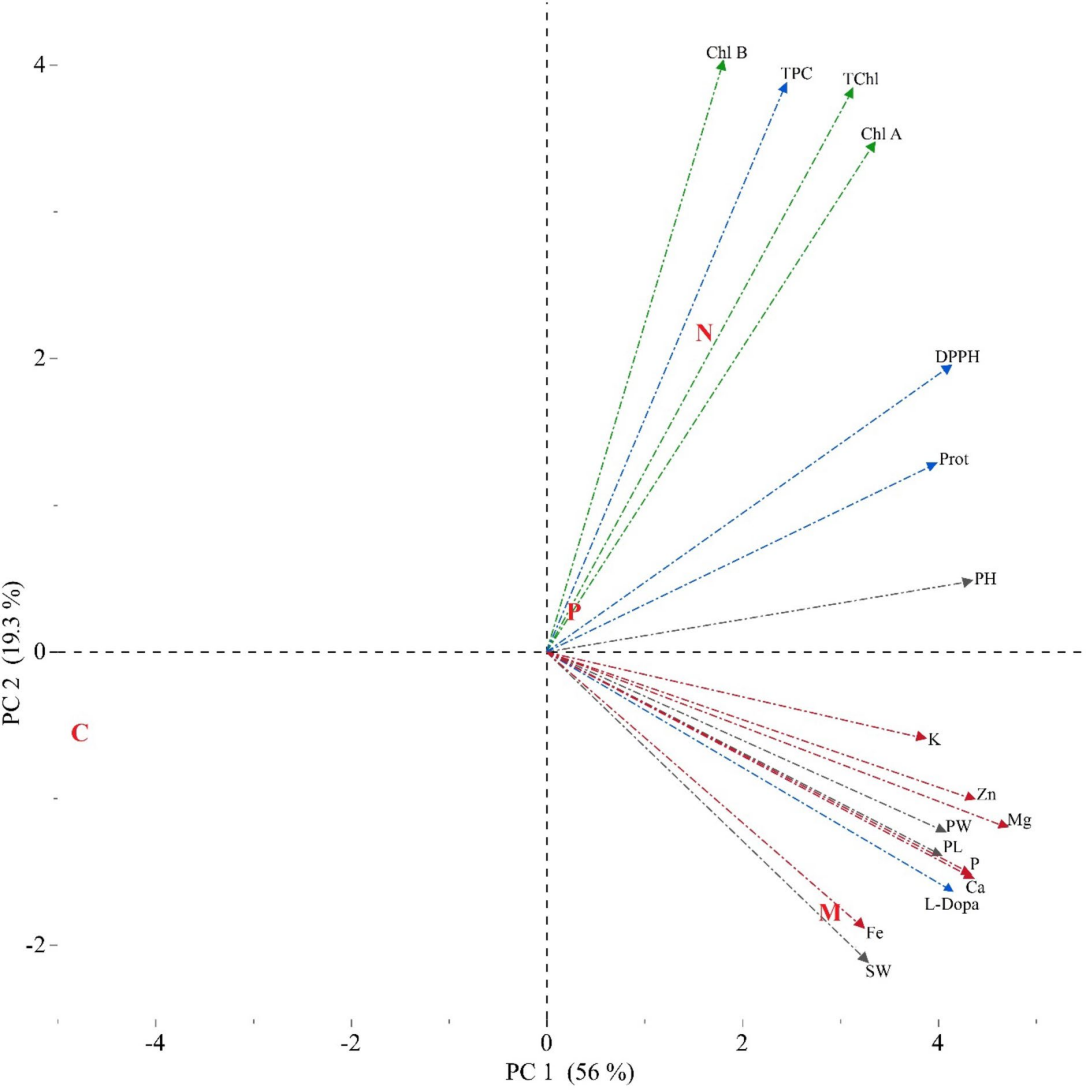
The biplot PCA analysis of the distribution of the AMF (M) and PGPR *Bacillus subtilis* (N), *Bacillus megaterium* (P) treatments and Control (C) with studied parameters (TPC: total phenol content, DPPH: total antioxidant capacity, Ca: calcium, K: potassium, Mg: magnesium, P: phosphorus, Fe: iron, Zn: zinc, PH: plant height, PL: pod length, PW: pod weight, Chl A: Chlorophyll a, Chl B: Chlorophyll b, TChl: Total chlorophyll).

Principal component analysis (PCA) explained 75.3% of the total variance caused by the first two components, PC1 accounting for 56% and PC2 for 19.3%. The PCA analysis separated control, AMF, *Bacillus subtilis*, and *Bacillus megaterium* treatments into different groups. The AMF inoculation was correlated with the elevated values of minerals and L-Dopa, and growth parameters. *Bacillus subtilis* was characterized by chlorophyll a, chlorophyll b, and total chlorophyll, and TPC contents. When all treatments were positively correlated to studied traits, the control possessed a negative relationship (Fig. 5).

### Correlations across traits

**Fig. 6:**
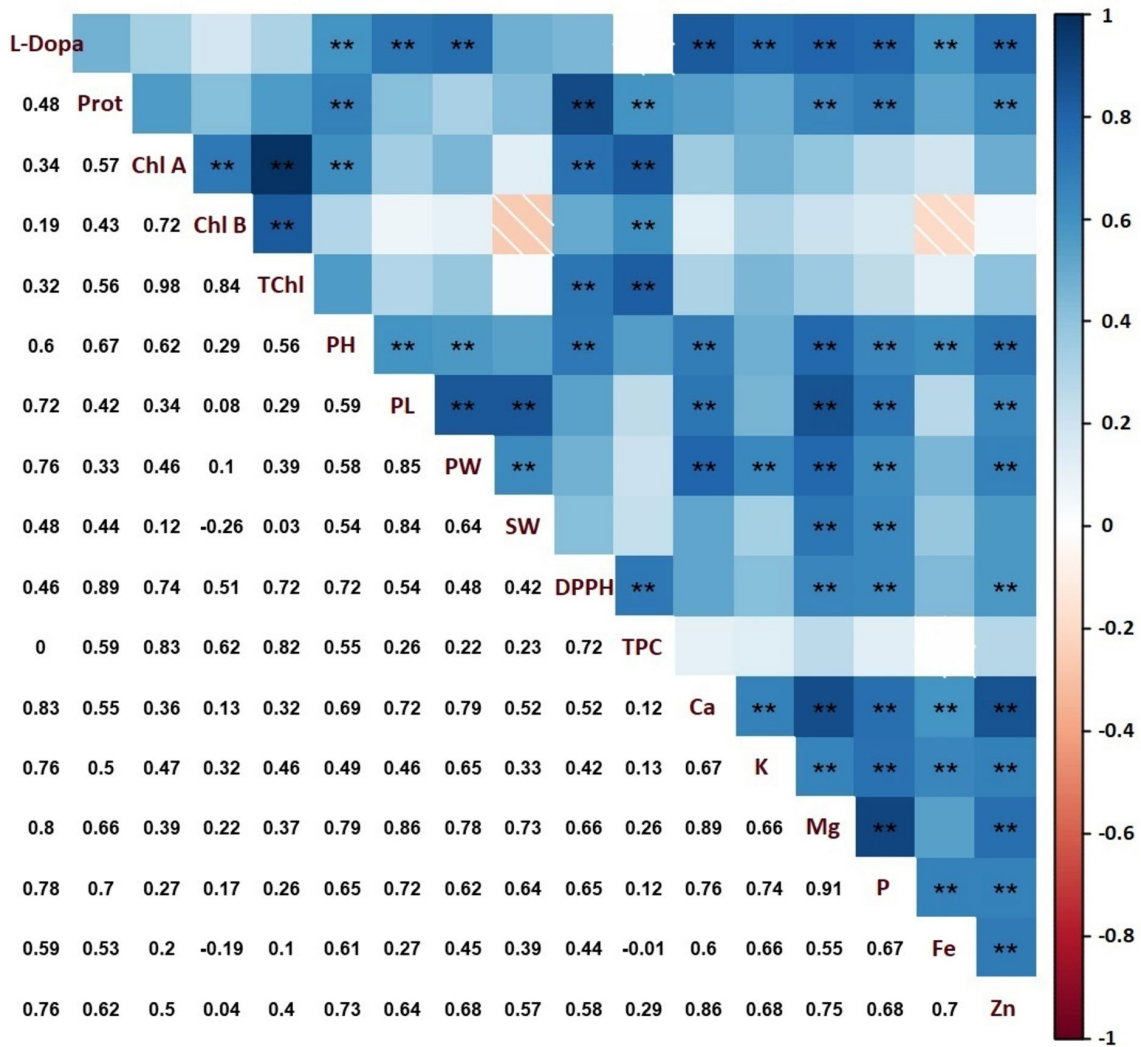
Correlations between the characteristics studied in faba bean. *, and ** indicates significance at *p* ≤ 0.05, *p* ≤ 0.01, respectively (TPC: total phenol content, DPPH: total antioxidant capacity, Ca: calcium, K: potassium, Mg: magnesium, P: phosphorus, Fe: iron, Zn: zinc, Prot: protein, PH: plant height, PL: pod length, PW: pod weight, SW: seed weight, Chl A: chlorophyll a, Chl b: chlorophyll b and TChl: total chlorophyll).

Positive correlations were found among most features analyzed in faba bean (Fig. 6). Magnesium and phosphorus exhibited the strongest correlation across minerals with a coefficient of *r* = 0.91. Only negative correlations were between chlorophyll b and seed weight (*r*=-0.26), chlorophyll b and Fe (*r*=-0.19), Fe and TPC (*r*=-0.01), which all were negligible. The correlation between Mg and all properties was significant at *p* ≤ 0.01, except for TPC, chlorophyll a, b, and total chlorophyll. Plant growth characteristics possessed positive significant correlations with minerals. Particularly, pod length exhibited the highest correlations. TPC and DPPH exhibited a strong positive correlation (*r* = 0.72). They also obtained significant positive correlations with chlorophyll and protein contents. L-Dopa was positively correlated to plant growth characteristics, TPC, and mineral elements content (*p* ≤ 0.01). Most minerals were positively correlated with growth characteristics but less correlated with seed weight.

## Discussion

In this study, AMF and PGPR applications enhanced plant height, pod length, and pod weight in faba beans compared to the control (Fig. 1; Table S1). The highest improvements were recorded in AMF-treated plants, followed by *Bacillus subtilis* and *Bacillus megaterium*. These findings align with previous reports indicating that AMF and PGPR enhance crop productivity and plant biomass^36–38^. Most studies have found that AMF and PGPR inoculation significantly increase plant height^37^, leaf number^38^, root biomass^39^, fruit quality^40^, chlorophyll content^41^, protein biosynthesis^42^, and soil enzymes^43^. AMF inoculation to faba bean plants resulted in an increase in pods per plant, seed weight per plant, pod weight per plant, and 100 seed yield^44^. PGPR and mycorrhiza inoculation of soybean induced the expression of genes involved in protein biosynthesis, and an increase in protein content^43^. Significant improvements in all growth characteristics by AMF and PGPRs in this study support previous reports on other plants and clarify the usefulness of these biostimulants in faba bean cultivation.

Chlorophyll a, chlorophyll b, and total chlorophyll content were significantly increased by biostimulant treatments, with *Bacillus subtilis* yielding the highest enhancement in this study (Fig. 2; Table S2). The positive effect of bacterial biostimulants on chlorophyll (a, b, and total) and photosynthesis rates in plant leaves may be related to increased N and P uptake^45^. Chlorophyll a contains a magnesium ion embedded within a large ring structure called chlorin. The presence of the magnesium center is what uniquely characterizes the structure as a chlorophyll molecule^46^. Chlorophyll is the green pigment found in chloroplasts, which are the sites for photosynthesis. Its primary function is to capture light energy from the sun and convert it, along with water and carbon dioxide, into glucose and oxygen. Plants use the glucose produced in this process for the growth of various plant parts^47^. In this regard, the enhanced chlorophyll and Mg contents supported by improved growth characteristics suggest a complete improvement in faba bean plants caused by AMF and PGPRs.

TPC accumulations were significantly higher in plants treated with *Bacillus subtilis*, with an increase of over three-fold compared to the control (Fig. 3; Table S3). On the other hand, L-Dopa was significantly higher in AMF-treated plants. The observed increase in L-Dopa content, which exceeded 2-fold in AMF-treated plants, highlights the potential of biostimulants to enhance bioactive secondary metabolites in faba beans. Similar findings were reported in some other plants, where PGPR treatments led to enhanced phenolic biosynthesis^48–50^. L-Dopa is a non-protein amino acid with a phenolic acid structure and its use as a dietary supplement in treating Parkinson’s disease has attracted global attention^10^. Approximately six million people worldwide suffer from Parkinson’s disease, and this number is predicted to double by 2040^51^. Faba beans have a significant L-Dopa concentration in their tissues^52^. L-Dopa content in green pods varied between genotypes, ranging between 4.16 mg and 54.29 mg kg^− 1^.^53^ The L-Dopa contents obtained in this study were in the range of previous reports and this study determined the L-Dopa shifts caused by AMF and PGPRs in faba beans for the first time. AMF inoculation led to more than a 2-fold increase in secondary metabolite content like L-Dopa, demonstrating the effectiveness of biostimulant treatment in enhancing the production of healthy compounds.

Plants can accumulate chemical compounds such as cyanogenic glycosides, glucosinolates, alkaloids, terpenes, and phenolics as a defense strategy in their tissues^54^. These secondary metabolites protect against biotic and abiotic stresses and act as scavengers of free radicals^55^. The exact mechanism of PGPR changing the phenolic profile in plants is unknown, but it is thought that PGPR plays a role in the synthesis of phenolic compounds in the plant^56^. Phenolic compounds and L-Dopa production increased in faba bean sprouts exposed to ultraviolet (UV), and secondary metabolite pathways were stimulated through abiotic stress^57^. Some studies have shown that PGPR can change the polyphenol composition in the root exudates of host plants and use these secondary metabolites as carbon sources^58–60^. Two pseudomonas PGPR strains in chickpea plants and Bradyrhizobia-arbuscular mycorrhiza inoculation in soybeans induced the accumulation of total phenols and phenolic acid synthesis^61,62^. The total phenol content in mature seeds of 14 genotypes varied between 10.12 mg^− 1^ GAE eq and 26.94 mg^− 1^ GAE Eq. ^63^ The TPC content in this study was similar to previous reports.

The findings of this study suggest that AMF and PGPR applications can serve as an alternative to synthetic fertilizers by enhancing nutrient uptake and plant growth. Reducing reliance on chemical fertilizers is crucial, as their continuous use leads to soil degradation and environmental pollution^64^. Biostimulants not only improve growth but also promote soil microbial activity, making them a sustainable alternative to conventional fertilization strategies. Biostimulants, a promising and environmentally friendly innovation, increase flowering, plant growth, fruit quality, crop productivity, and resistance to various abiotic stressors^65^. Plant growth-promoting rhizobacteria (PGPR) and arbuscular mycorrhizal fungi (AMF), sometimes classified as biofertilizers, increase nutrient uptake by increasing root area and biomass and promote plant growth through many mechanisms^66^. Many studies have recently shown that AMF and PGPR biostimulants promote plant growth and nutrient uptake in *Vicia faba*^67–70^, *Phaseolus vulgaris*^71–74^, *Cicer arietinum*^36,75,76^, and *Glycine max*^77,78^. Faba beans are a rich source of protein and non-nutritional secondary metabolites that improve nutritional quality in diets and benefit human health^79^. It has recently attracted interest as a functional food source due to its high concentration of phenolics, flavonoids, terpenoids, lignans, non-protein amino acids, and bioactive phytochemicals such as L-Dopa that contribute to health^5^.

DPPH radical scavenging activity was significantly enhanced in biostimulant-treated plants, with Bacillus subtilis application exhibiting the highest antioxidant potential (Fig. 3; Table S3). The PCA analysis (Fig. 5) further supports this trend, as DPPH activity is closely associated with the biostimulant-treated groups, particularly Bacillus subtilis and AMF, which showed a strong separation from the control group along PC1. This indicates that the observed increase in antioxidant capacity is highly correlated with biostimulant applications. Moreover, the correlation matrix (Fig. 6) reveals a positive association between total phenolic content TPC and DPPH activity, suggesting that the enhanced phenolic compound biosynthesis contributed to the increased antioxidant potential. Similar findings have been reported in basil and coriander, where biostimulant applications led to significant improvements in phenolic content and antioxidant activity^29,37^.

Protein content was significantly increased in biostimulant-treated faba bean pods compared to the control (Table S3). The PCA analysis (Fig. 5) highlights a clear separation of biostimulant-treated groups, indicating that protein accumulation is strongly associated with these treatments. The increase in protein content is likely due to the improved nitrogen uptake facilitated by AMF and PGPR. Previous legume crop studies demonstrated that PGPR and AMF can enhance protein biosynthesis^78,80^. The strong correlation between protein content and phosphorus levels observed in the correlation matrix (Fig. 6) further supports this hypothesis. Thus, the observed increase in protein content in this study suggests that these treatments may be an effective strategy for improving the nutritional quality of legume crops.

The mechanism by which PGPR and AMF stimulate plant growth is not yet fully understood, but it is assumed that they support the plant in the production of plant hormones and increase plant nutrient uptake, solubilization, uptake and transport of mineral substances such as phosphorus and nitrogen in the rhizosphere^81^. The AMF symbiosis creates colonized mycelium in the root, and the hyphae expand the effective absorption surface^82^. The increase in phosphorus in the plant may be related to the release of organic acids by AMF and PGPR, the phosphatase enzyme, or the increase in phosphorus availability in the soil by chelating phosphate-bound cations^83,84^. *Bacillus megaterium* and *Bacillus subtilis* can hydrolyze organic phosphate in the soil by producing phytase, increasing the concentrations of inorganic phosphorus in the plant^85–87^. Phosphorus is known for its necessity fruiting in plants. In this study, the phosphorus content was significantly improved by AMF and PGPRs, and it was positively correlated to growth characteristics (Fig. 4; Table S4; Fig. 6). Therefore, biostimulants were evaluated as an outstanding tool for improving the growth of faba beans.

AMF help plants to uptake nutrients with low mobility or poorly soluble in the soil^88^. Among the immobile elements, potassium is one of the most abundant elements in the soil composition, but its water-soluble forms are present in very low (0.1–0.2%) amounts^89^. As a result of the symbiotic relationship with the mycorrhizal fungi, the hyphae formed in the plant root significantly enhance potassium uptake^90^. PGPR and AMF can produce siderophores in the rhizosphere and low-molecular-weight organic compounds (organic acid, phenols, secondary compounds) with a high affinity for binding to metal ions^91^, chelating Fe^+ 2^, Mg^+ 2^, Ca^+ 2^ and Zn^+ 2^ ions, and improving their bioavailability^92^. The improvement of mentioned nutrients was detected in plants such as sorghum^93^, wheat^94,95^, soybean^59^ and black cumin^96^. Here, we also report significant improvements in the amounts of K, Fe, Zn, Ca, and Mg by biostimulants, particularly by AMF (Fig. 4; Table S4).

## Conclusion

This study evidenced that arbuscular mycorrhizal fungi (AMF) and plant growth-promoting rhizobacteria (PGPR) significantly enhanced the morphological characteristics, bioactive compounds, and mineral nutrient content of faba bean pods. Notably, this research marks the first to explore the impact of biostimulants on the L-Dopa compound, a critical therapeutic agent for Parkinson’s disease, revealing a remarkable increase under AMF and PGPR treatments. The improved nutritional profile, particularly the increase in essential minerals, further underscores the potential of these biostimulants in promoting biofortification. These findings demonstrated that AMF and PGPRs are promising tools for developing biofortified faba beans, contributing to sustainable agriculture and improving human health. Future research should further investigate the long-term impacts of these biostimulants on other legume species and evaluate their scalability across diverse ecological settings, ensuring broader applications in biofortified crop production.

## Electronic supplementary material

Below is the link to the electronic supplementary material.

Supplementary Material 1

## Data Availability

The data presented in this study are available upon request from the corresponding author.
