# Supplementary material for: Enhancements in morphology, biochemicals, nutrients, and L-Dopa in Faba bean through plant growth promoting rhizobacteria and arbuscular mycorrhizal Fungi
Source: Sci Rep. 2025 Mar 3;15:7390. doi: 10.1038/s41598-025-92486-8 (PMC11876452; doi:10.1038/s41598-025-92486-8)
Supplement: Supplementary file 1 — Supplementary Material 1 [file 41598_2025_92486_MOESM1_ESM.docx]

**Enhancements in Morphology, Biochemicals, Nutrients, and L-Dopa in Faba Bean Through Plant Growth-Promoting Rhizobacteria and Arbuscular Mycorrhizal Fungi**

**Supplementary Materials**

**Table S1.** Alteration in growth parameters by the treatments in faba beans

| Treatments | Plant height (cm) | Pod length (cm) | Pod weight (g) | Seed weight (g) |
| --- | --- | --- | --- | --- |
| Control | 121.93 ± 8.75 b | 13.03 ± 0.82 c | 25.33 ± 4.72 c | 3.57 ± 0.65 a |
| AMF | 138.33 ± 3.51 a | 14.77 ± 0.37 a | 35.41 ± 2.87 a | 4.34 ± 0.32 a |
| *Bacillus subtilis* | 137.67 ± 2.08 a | 14.44 ± 0.27 ab | 31.88 ± 2.19 ab | 4.16 ± 0.14 a |
| *Bacillus megaterium* | 135.00 ± 3.61 a | 13.53 ± 0.67 bc | 28.25 ± 1.88 bc | 4.12 ± 0.51 a |

Different letters in the same column indicates difference at p≤0.05 according to LSD test.

**Table S2.** Chlorophyll (a, b, and total) contents according to the treatments in faba beans

| Treatments | Chlorophyll a  (mg g^-1^ F.W) | Chlorophyll b  (mg g^-1^ F.W) | Total chlorophyll  (mg g^-1^ F.W) |
| --- | --- | --- | --- |
| Control | 1.91 ± 0.04 c | 0.84 ± 0.17 b | 2.75 ± 0.26 b |
| AMF | 2.40 ± 0.08 b | 0.95 ± 0.05 ab | 3.35 ± 0.16 b |
| *Bacillus subtilis* | 3.10 ± 0.15 a | 1.19 ± 0.18 a | 4.29 ± 0.24 a |
| *Bacillus megaterium* | 2.37 ± 0.39 b | 0.96 ± 0.12 ab | 3.33 ± 0.35 b |

Different letters in the same column indicates difference at p≤0.05 according to LSD test.

**Table S3.** The changes in bioactive compounds by the treatments in faba beans

| Treatments | DPPH (%) | L-Dopa (µg mL^-1^) | Protein (%) | Total phenol (mg GAE^-1^ g^-1^) |
| --- | --- | --- | --- | --- |
| Control | 28.21 ± 1.56 b | 12.18 ± 0.88 d | 20.22 ± 0.84 b | 16.34 ± 0.60 c |
| AMF | 53.63 ± 7.58 a | 23.36 ± 1.22 a | 22.79 ± 0.17 a | 24.14 ± 3.82 c |
| *Bacillus subtilis* | 64.32 ± 10.87 a | 16.70 ± 0.67 b | 23.05 ± 1.01 a | 53.80 ± 6.32 a |
| *Bacillus megaterium* | 57.23 ± 1.67 a | 14.56 ± 0.94 c | 23.24 ± 0.48 a | 39.43 ± 6.22 b |

Different letters in the same column indicates difference at p≤0.05 according to LSD test.

**Table S4.** Mineral nutrient content of faba beans affected by biostimulants treatments

| Treatments | Ca (mg 100g^-1^) | Fe (mg 100g^-1^) | K (mg 100g^-1^) | Mg (mg 100g^-1^) | P (mg 100g^-1^) | Zn (mg 100g^-1^) |
| --- | --- | --- | --- | --- | --- | --- |
| Control | 120.67 ± 5.51 c | 4.77 ± 0.42 b | 427.33 ± 63.34 b | 97.00 ± 2.65 c | 647.50 ± 5.00 c | 2.82 ± 0.14 b |
| AMF | 161.33 ± 9.07 a | 6.57 ± 0.21 a | 638.33 ± 21.22 a | 119.00 ± 4.58 a | 715.83 ± 10.41 a | 3.51 ± 0.26 a |
| *Bacillus subtilis* | 140.67 ± 9.07 b | 5.16 ± 0.56 b | 525.33 ± 58.96 ab | 111.67 ± 5.69 ab | 682.50 ± 15.21 b | 3.26 ± 0.14 a |
| *Bacillus megaterium* | 136.00 ± 4.58 b | 6.38 ± 0.84 a | 548.00 ± 97.37 ab | 109.00 ± 4.00 b | 695.00 ± 23.85 ab | 3.20 ± 0.09 a |

Different letters in the same column indicates difference at p≤0.05 according to LSD test.
